# What do we see in pictures? The sensory individuals of picture perception

**DOI:** 10.1007/s11098-022-01864-9

**Published:** 2022-08-23

**Authors:** Bence Nanay

**Affiliations:** Centre for Philosophical Psychology, University of Antwerp, D 413, Grote Kauwenberg 18, Prinsstraat 13, 2000 Antwerp, Belgium

**Keywords:** Sensory individuals, Perceptual representation, Picture perception, Spatiotemporal regions

## Abstract

When I am looking at an apple, I perceptually attribute certain properties to certain entities. Two questions arise: what are these entities (what is it that I perceptually represent as having properties) and what are these properties (what properties I perceive this entity as having)? This paper is about the former, less widely explored, question: what does our perceptual system attribute properties to? In other words, what are these ‘sensory individuals’. There have been important debates in philosophy of perception about what sensory individuals would be the most plausible candidates for which sense modalities. The aim of this paper is to ask a related question about picture perception: what is the sensory individual of picture perception? When we look at a picture and see an apple depicted in it, what kind of entity do we see? What do we perceptually attribute properties to? I argue that the most straightforward candidates (ordinary objects, sui generis sensory individuals, no sensory individuals) are all problematic and that the most plausible candidate for the sensory individuals of picture perception are spatiotemporal regions.

## Introduction: sensory individuals

We perceive things around us as having various properties. I will use what I take to be a harmless bit of terminology and talk about this in representational terms: we perceptually represent certain entities as having certain properties; we perceptually attribute certain properties to certain entities. Two questions arise: what are these entities and what are these properties? This paper is about the first one of these questions: What does our perceptual system attribute properties to?

Not all properties that we represent objects as having are perceptually represented. I may represent an apple as having the property of being a granny smith apple picked by Mr. Taylor in Oregon in 2022, but this is not a property that is likely to be perceptually represented. A structurally similar problem arises when we try to understand what entities these properties are perceptually attributed to. We may attribute properties non-perceptually to everyday objects, like the apple or the cedar tree in front of my window, but the question is what we *perceptually* attribute properties to. As the range of properties attributed perceptually and non-perceptually are often not the same, the entities these properties are attributed to may not be the same either.

To avoid confusion, I call the entities our perceptual system attributes properties to ‘sensory individuals’ (Cohen, 2004). Sensory individuals are not necessarily what we take ourselves to be perceiving (as perceptual content is not directly introspectable). Nor are they necessarily conscious. Whenever we have a perceptual representation, conscious or unconscious, this perceptual representation attributes properties to something. This something is what I call ‘sensory individual’ and it may be different from what our perceptual reports are about.

Why should we posit sensory individuals at all? The widely cited reason is that properties need to be bound to particulars in perception. To use the classic example (Jackson, 1977), if I see a red triangle and a blue square, the property of being red and of being a triangle are bound to one individual, whereas the property of being blue and the property of being a square are bound to the other. If they were not bound, then this perceptual state would be indistinguishable from seeing a red square and a blue triangle. Note that this consideration only specifies that the perceptually attributed properties need to be bound to an individual but there is more than one candidate for what this individual may be.

This paper is about the sensory individuals of a very specific kind of perceptual phenomenon: picture perception. And picture perception has an extra layer of complication when it comes to sensory individuals. When we see an apple in front of us, we perceptually attribute properties to it. When we see an apple in a picture, in some sense we see not one but two things: the picture surface and the apple depicted by the picture.

The nature of this twofold perceptual state has been an important theme in the picture perception literature (starting with Wollheim 1980, see also Lopes 1996, Nanay, 2004, 2005), but what matters for us is that we can’t just take it for granted that an account of sensory individuals for vision will automatically work as an account of sensory individuals for picture perception, because in picture perception, there are two sensory individuals, corresponding to the two folds of picture perception, the perception of the surface and the perception of the depicted object. We could just plug in our best theories of sensory individuals when it comes to the perception of the picture surface, after all, the picture surface is right there in front of us. But things get more complicated when it comes to the sensory individual of what we see in the picture. The hope is that by understanding this more complicated question concerning the sensory individuals of picture perception, we can also make progress with regards to the sensory individuals of perception per se.

In Sect. 2, I give an overview of the most important accounts of sensory individuals in various sense modalities and then in Sect. 3, I show that none of these standard candidates (ordinary objects, sui generis sense modality-specific sensory individuals, no sensory individuals) work when it comes to the sensory individuals of picture perception. In Sects. 4 and 5, I argue that the sensory individuals of picture perception are spatiotemporal regions and in Sect. 6, I trace the consequences of this view back to the more general question about face to face perception.

## Varieties of sensory individuals

The plausibility of an account of sensory individuals depends on which sense modality we talk about. The mainstream view of the sensory individuals of vision is that they are ordinary objects like an apple or a cedar tree. As David Armstrong says, “In perception, properties and relations are attributed to objects” (Armstrong, 2004, p. 20, see also Shoemaker, 1990, p. 97, Brewer, 2007, p. 88 to mention just a few examples). Just what would count as an ordinary object is debated, but the most empirically plausible view seems to be that an ordinary object is a “spatio-temporally confined and continuous entity that can move and take its features with it” (Matthen, 2005, p. 281, see also Pylyshyn, 2007, Cohen, 2004, Matthen, 2004, 2010, 2021 for similar views). There are some dissenting voices, who argue that sensory individuals of vision are spatiotemporal regions—I will come back to this view in Section IV below. I briefly discuss the debates about the sensory individuals of non-visual sense modalities as some of the proposed accounts in these non-visual sense modalities will play an important role in the discussion about potential candidates for the sensory individuals of picture perception (and for this reason, I will focus on audition and olfaction only, setting aside tactile perception).

The debate about the sensory individuals of audition is very different from the “ordinary object” versus “spatiotemporal region” debate concerning the visual sense modality (Matthen, 2010; O’Callaghan, 2008a). The main candidate for the sensory individuals of audition are sounds, both historically (with support from Aristotle, Berkeley, Strawson, and Warnock) and in the contemporary literature (Nudds, 2010, O’Callaghan, 2008b, p. 318, 2011, p. 609, Bregman, 1990, Martin, 1997). As Mohan Matthen summarizes, “it is the sound of the coach that is directly heard, not the coach itself” (Matthen, 2010, p. 78, see also Young & Nanay, 2022).

The debate about the sensory individuals of audition is very different from the one about the sensory individuals of vision inasmuch as here the main question is not what we perceive, but rather, what we perceive directly or, as O’Callaghan says, what we perceive ‘in the first instance’ (O’Callaghan 2011, p. 609). Do we perceive ordinary objects ‘in the first instance’, or do we perceive ordinary objects in virtue of perceiving sounds (that is, we perceive sounds ‘in the first instance’)?

In the case of the olfactory sense modality, we get a similar picture. The mainstream view here is that we smell odors—a claim that would be equivalent to saying that we hear sounds: “smells […] represent the environmental entities by representing odors” (Lycan 1996, p. 148, see also Lycan, 2000, see also Batty, 2010, 2011, on this debate). As the Lycan quote shows, here, like in the case of the auditory sense modality, the main question is whether we perceive ordinary objects ‘in the first instance’ or we perceive ordinary objects in virtue of perceiving odors.

When it comes to olfaction, however, there is yet another option, which I will come back to in the context of picture perception. This alternative is that olfaction does not attribute properties to an individual at all. It represents properties without representing any particular individual as having these properties. This would imply that there are no sensory individuals at all in the case of olfaction. Without endorsing this view, I want to point out that it is not a crazy view. The main consideration in favor of sensory individuals, that is, in favor of the claim that properties are bound to particulars was Jackson’s argument about how seeing a red triangle and a blue square is different from seeing a red square and a blue triangle. And it has been argued that we have no reason to suppose that similar considerations apply in the case of the olfactory sense modality (Batty, 2010, 2011, see also Keller, 2017). Again, I am not sure that these arguments are convincing, but if they are, that is, if we have no reason to suppose that there are sensory individuals in the olfactory sense modalities, then we may be justified at reviving some version of the long (and maybe unfairly) abandoned adverbialist accounts of perception (Tye, 1989), but restricting them to the olfactory sense modality only. As the most commonly cited argument against adverbialism is Jackson’s argument about the red triangle and the blue square, if it does not apply in the case of the olfactory sense modality, then adverbialism, as long as it is restricted to olfaction, seems to be a plausible candidate.

The question I want to examine in this paper is about the sensory individuals of picture perception. As we have seen in this section, there is no shortage of potential candidates for sensory individuals. And it is not to be taken for granted that because the sensory individuals of vision (according to the mainstream account) are ordinary objects, this account generalizes to the other sense modalities. Similarly, we should not take it for granted that because the sensory individuals of vision (according to the mainstream account) are ordinary objects, this account generalizes to picture perception.

## Picture perception

The aim of this section is to show that none of the obvious candidates for sensory individuals are plausible as the sensory individuals of picture perception. We have seen in the last section that according to the mainstream accounts, the sensory individuals of vision are ordinary objects and the sensory individuals of audition and olfaction are sounds and odors, respectively – sensory individuals specific to the sense modality in question. We have also seen a possible view in the case of olfaction, according to which in olfaction, there are no sensory individuals. So the obvious candidates for the sensory individuals of picture perception would be (i) ordinary objects, (ii) some kind of sui generis sensory individuals, and (iii) no sensory individuals. I will argue that none of these are particularly promising when it comes to picture perception before I give a positive account of the sensory individuals of picture perception.

Some preliminaries: I will assume throughout this paper that picture perception is a genuine case of perceiving. When we look at a picture, we do indeed perceptually represent the depicted scene (by which I merely mean depicted sensory individual here). Even those who want to insist that we do not strictly speaking see the depicted scene (because, for example, the relevant causal link that is necessary for seeing is missing) could go along with the weaker claim, which is all I need for the purposes of this paper, namely, that when we look at a picture, we perceptually represent the depicted sensory individual. It is not the case, for example, that we merely imagine the depicted object (or maybe imagine our experience of the picture surface to be an experience of the depicted object, see Walton 1990). I will not rehearse arguments against these imagination-based views of picture perception here (but see Wollheim, 2003, Nanay, 2021a).

With these preliminaries out of the way, let’s consider the most straightforward option: ordinary objects.

### Ordinary objects

The sensory individuals of vision, according to the mainstream view, are ordinary objects. So it would be natural to extend this account to the form of visual perception that is picture perception (although the literature on tactile pictures may complicate this move, see Voltolini & Calzavarini, 2021). This would amount to the claim that when we look at a picture of an apple, we perceptually attribute properties (of, say, being red and being round) to the depicted ordinary object: the apple.^1^

Sounds simple enough, but I will argue that this proposal is problematic for a number of reasons. First, there are two possible versions of the ordinary object view (both in the case of vision per se and in the case of picture perception) depending on whether ‘ordinary objects’ are taken to be particulars or object types. I assume that the standard version of the ‘ordinary objects’ view is that these are particulars (and this has been an explicit assumption of the defenders of this view I cited in Sect. 2 above) but for the sake of covering all options, I will come back to the possibility that the sensory individuals of picture perception are object types in Sect. 3.2 below. If the sensory individuals of vision are particulars, and not object types, then seeing two indistinguishable IKEA chairs amounts to perceptually attributing properties to two different particulars. The content of my perceptual state when seeing two indistinguishable IKEA chairs are different because the particular (the sensory individual) the properties are attributed to is different.

The most straightforward way of extending the standard account of sensory individuals of vision to picture perception would be to say that when I see an apple in a picture, I attribute properties to a particular apple. Now some pictures arguably do represent particulars. Photographs could be thought to represent particulars. If I take a snapshot of an IKEA chair, then this snapshot is, in some sense, about this particular chair and not of any other, indistinguishable chairs. One way of cashing this out is that the causal chain that leads to the creation of the photograph ties the picture to this particular and not to any other. Portraits could also be thought to represent particulars: if the painter paints a portrait of one of two identical twins, it is the portrait of this particular person and not her twin sister. In this case, it is the artist’s intention that is usually credited with picking out one particular (and not others) as the depicted object.

While some pictures (like photographs or portraits) depict concrete particulars, others don’t. They depict object (or persons) of a certain kind (see Wollheim 1987 for a classic analysis). So if our picture perception attributes properties to any particulars, it systematically misrepresents. But let us return to those pictures that do represent concrete particulars. Even in this case, it is extremely questionable whether the particularity of the depicted object is perceptually represented. Remember the two main candidate for what makes some pictures depict particulars: causal link (for example, in the case of photographs) and the artist’s intention (in the case of portraits). Neither of these seem to be straightforwardly perceptually represented. While there is a debate about whether we can perceive causation both in philosophy and in psychology (see, e.g., Siegel, 2009), this discussion is about whether we can perceive causation between two objects that we do perceive. Note that what would be required for the particularity of depicted objects to be perceived is something much stronger: the perceptual representation of a causal link, where one of the causal relata is not perceived at all. Second, while there is, again, a debate about whether we can perceive someone’s intentions when looking at their face (see, e.g., Caruso et al., 2010), what would be required for the particularity of depicted objects to be perceived is something much stronger: the perceptual representation of someone’s intention without seeing their face (and, usually, without ever having seen them).

For all these reasons, the ordinary object view is not particularly promising when it comes to the sensory individuals of picture perception (see also Wollheim 1987, pp. 67–71, Zeimbekis, 2010 and Aasen, 2016 for related arguments). One may object that my assumption that ordinary objects are to be understood as particulars was not justified. I will consider views that don’t make that assumption in the next subsection.

### Sui generis sensory individuals

Simply extending an account of the sensory individuals of vision, that is, of ordinary objects as sensory individuals to picture perception does not seem to work very well. But as we have seen when discussing the non-visual sense modalities, this is not the only option. According to the mainstream view, the sensory individuals of audition are sounds—sui generis sensory individuals specific to the auditory sense modality. Similarly, the sensory individuals of olfaction are widely held to be odors—sui generis sensory individuals specific to the olfactory sense modality. The suggestion then would be that picture perception has its own sui generis sensory individuals.

One relatively small change one could make when moving from vision in general to picture perception would be to claim that while the sensory individuals of vision are particular token ordinary objects, the sensory individuals of picture perception are types of ordinary objects, or, in short, object-types. (This strategy would also be available to those who would question the starting assumption of the previous section, namely, that ordinary objects as the sensory individuals of vision are understood as particular token ordinary objects.)

So when we see an apple in a picture, we don’t see a particular apple, we just see an apple-type. But apples (like everything else) can be typed or individuated in a variety of ways. If the sensory individual of picture perception is an object-type, what kind of type is it? Given that the perceptual system will be insensitive to some ways of typing apples, such as whether they were picked by Mr. Taylor in Oregon in 2022, the natural answer would be to say that object-type in question is a perceptual object-type: objects that are perceptually similar to the apple I see. In short, apples that have a certain visual appearance. The proposal is then that the sensory individuals of picture perception are objects with a certain visual appearance.

Picture perception, according to this proposal, would attribute properties to an object-type. Now, it is clearly possible in general to attribute properties to object-types. We can, for example, attribute properties to object-types by means of using language. When I say that mammals are vertebrates, this is an attribution of properties to object-types. But it is much less clear that properties can be attributed to object-types perceptually.

Here is the problem with the perceptual attribution of properties to object-types. Object-types are individuated by means of having certain properties—in the perceptual case, by means of having observational properties. So attributing a property (of, say, being circular) to an object-type would amount to attributing a property (of, say, being circular) to, say, a red-looking object. But given that this object-type is individuated by means of having the property of being red-looking, just having this object-type as sensory individual itself presupposes the attribution of a property, namely, the property that individuates the type (in my example, the property of being red-looking). But then we are back with the original question, namely, the question about what sensory individual this (object-type individuating) property is attributed to. And, as the argument from the previous section shows, it can’t be a particular. I will return to the option that this (object-type individuating) property is not attributed to anything (that they are free-floating uninstantiated universals) in Sect. 3.3 below.

But there are other candidates for sui generis sensory individuals in the case of picture perception. I will use audition as a parallel case in developing what I take to be the most straightforward accounts of such sui generis sensory individuals. Again, according to the standard account, audition also has sui generis sensory individuals: sounds. And we can use the various ways of thinking about sounds (and also odors) as a parallel case.

There is no agreement about what sounds are supposed to be. Some take sounds to be particulars (Casati & Dokic, 1994; O’Callaghan, 2007) and some take them to be qualities of particulars (Kulvicki, 2008; Leddington, 2019). Those who take sounds to be particulars claim that they are the vibration-producing particular events or maybe disturbance events close to the sound-producing event or maybe abstract particulars. Either way, they are particular events. If we apply this way of thinking about the sensory individuals of audition (sounds) to the domain of picture perception, what we get is that the sensory individuals of picture perception are some kind of particulars either corresponding to, or located in spatial proximity to, the depicted object or event. But in this case, all the arguments in the previous subsection would apply against any such account as this would imply that the sensory individuals of picture perception are depicted particulars and I argued against this view in the previous section.

Some proponents of the view that sounds are the sensory individuals of audition take sounds to be qualities of particulars, rather than particulars. Opinions differ about just what kinds of qualities these are, one important option being stable dispositional properties (Kulvicki, 2008). If we transfer this way of thinking about the sensory individuals of audition (sounds) to the domain of picture perception, what we get is that the sensory individuals of picture perception are the properties of particulars (see Wollheim 1987, Zeimbekis, 2010, Martin, 2012 for suggestions along these lines).

There are two options. These properties of particulars may be tropes (particularized properties that cannot be instantiated twice) or they may be universals. If they are tropes, then, given that tropes are particulars, the argument from the previous section would apply to this view. If they are universals, then they are instantiated universals—they are instantiated by the object they are the properties of. And given that our visual system is sensitive to very specific properties, we can say that the sensory individuals of picture perception are observational properties (understood as universals) of particulars.

So the view is that the observational properties are themselves the sensory individuals of picture perception. According to this account, picture perception would amount to the perceptual attribution of properties to properties. The parallel view has been explored in the case of audition (Kulvicki, 2008; Leddington, 2019), where pitch (a property) is attributed to a sound (also thought of as a property), and in the case of vision hue and saturation (observational properties) are often taken to be attributed to color (also an observational property). I want to set aside the worry that this would entail a highly complex mental process of attributing a property-type to another property-type and ask a logically prior question: is the observational property that serves as a sensory individual (that other observational properties are attributed to) instantiated or uninstantiated?

I will come back to examining uninstantiated universals in the next subsection. But given that the view here is that the sensory individual is the observational property of a particular, this property is instantiated. But if so, then we are back with the original question about what they are attributed to (and, again, the most natural answer that they are attributed to a particular is not an option).

### No sensory individuals

In the previous section, I outlined ways of applying accounts of the sensory individuals of audition (sounds) to the domain of picture perception and argued that they don’t work. In this section, I will explore whether we can make a move in the context of picture perception that has been made in the debate about the sensory individuals of olfaction. As we have seen, not everybody agrees that olfaction has sensory individuals. An alternative would be to argue that olfaction represents uninstantiated universals. These universals are not attributed to anything—thus there are no sensory individuals of olfaction.

A similar move could be and has been made when it comes to the sensory individuals of picture perception. Given that the view that properties are attributed to particulars in picture perception is not too promising, we could settle for the view that properties are not attributed to anything in picture perception (Aasen, 2016 considers this option, which she calls the Platonian universals view and she prefers it to the instantiated universals view).

Comparing this view to the structurally similar account of olfaction is instructive. In the case of olfaction, this ‘no sensory individual’ view is motivated by the argument that the so-called ‘Many Properties Problem’ fails to apply in the case of olfaction. The ‘Many Properties Problem’ was initially an argument against adverbialism and it is supposed to be the very reason why we talk about sensory individuals to begin with. Attributing the property of being red to a square and the property of being blue to a triangle is different from attributing the property of being red to a triangle and the property of being blue to a square. The first representation amounts to a red square and a blue triangle, whereas the second representation amounts to a red triangle and a blue square. Very different representations. And that is one of the reasons why we need to postulate a sensory individual, to which properties are attributed.

It has been argued, however, that in the case of olfaction, the Many Properties Problem fails to arise. Olfactorily attributing the olfactory property of being pungent to the pizza and the olfactory property of being burnt to the garlic bread is not different from olfactorily attributing the olfactory property of being burnt to the pizza and the olfactory property of being pungent to the garlic bread. I am not endorsing this argument, but if this argument were correct, then we would lose the arguably most important reason why sensory individuals would need to be postulated in the case of olfaction. And it is at least not a wildly implausible claim that the Many Property Problem does not arise in olfaction, as olfaction binds smells differently (if at all) from the way vision binds observational properties (I want to leave open the question about whether we can make a straightforward inference from the non-applicability of the Many Property Problem to the conclusion that there is no need to posit sensory individuals in olfaction).

Crucially, the Many Properties Problem does arise in the case of picture perception (whether or not it arises in the case of olfaction). Looking at a picture of a red square and a blue triangle is very different from looking at a picture of a red triangle and a blue square. But if picture perception had no sensory individuals, then these two instances of picture perception would amount to the same thing. In short, the ‘no sensory individuals’ view can’t explain how observable properties are bound together in the case of picture perception.

## Spatiotemporal regions

I will argue that the sensory individuals of picture perception are not the standard candidates from the visual, auditory or olfactory sense modality. The most plausible way of thinking about the sensory individuals of picture perception is as spatiotemporal regions. Spatiotemporal regions have been proposed as possible sensory individuals of vision, but in that debate, this is very much a minority view. And spatiotemporal regions don’t even register in the debates about the sensory individuals of audition or olfaction (see Cohen, 2010 and Nanay, 2013 for two rare exceptions, albeit both quite noncommittal).

So, let’s go back to vision in general, bracketing picture perception for now. In this Section, I argue that the spatiotemporal region view of visual sensory individuals is not as hopeless as it has been assumed. In the next section, I will apply this view to picture perception.

As we have seen, the standard view in the case of vision is that we perceive ordinary objects. When I see an apple as red, I perceptually attribute the property of being red to the apple (Armstrong, 2004, p. 20, see also Shoemaker, 1990, p. 97, Brewer, 2007, p. 88, Matthen, 2005, p. 281, see also Pylyshyn, 2007, Cohen, 2004, Matthen, 2004, 2010 for similar views).

But here is another option when it comes to the sensory individuals of vision: spatiotemporal regions. The main champion of the alternative, minority, view is Austen Clark, who characterizes sensory individuals (which he calls ‘phenomenal individuals’) as “regions or volumes at which qualities seem to be located” (Clark, 2000, p. 61).^2^ To put it differently, according to Clark, our perceptual system attributes properties to places, not ordinary objects. As he says, "the sensation of a red triangle […] picks out places and attributes features to them" (Clark, 2000, p. 147, see also Clark, 2004 for clarifications). Spatiotemporal regions are regions in time–space. They are not particulars. Particulars are located at a spatiotemporal region, but they ate not identical to the spatiotemporal region they are located at.^3^

The first thing to note is that the spatiotemporal view solves the Many Properties Problem as much as the ordinary object view does. Seeing a red square on the left and a blue triangle on the right is different from seeing a red triangle on the left and a blue square on the right. And the spatiotemporal view can explain this difference in terms of properties attributed to the spatiotemporal region on the left and the spatiotemporal region on the right. In the first case, the properties of being red and being square-shaped are attributed to the spatiotemporal region on the left and the properties of being blue and being triangle-shaped are attributed to the spatiotemporal region on the right. And in the second case, the properties of being red and being triangle-shaped are attributed to the spatiotemporal region on the left and the properties of being blue and being square-shaped are attributed to the spatiotemporal region on the right. Clearly very different perceptual episodes.

The spatiotemporal regions view has been dismissed for the following three reasons:

First, spatiotemporal regions view is taken to be too revisionary, that is, too revisionary of our everyday conception of perception. We take ourselves to be perceiving ordinary objects: tables, water bottles, trees. If the spatiotemporal regions view is correct, we’re always wrong about what we perceive (Cohen, 2004, p. 476, see also Matthen, 2012).

Second, the spatiotemporal regions view is taken to have problems accounting for the perception of movement. As Susanna Siegel says: “What happens in sensory phenomenology when a subject sees a basketball make its way from the player's hands to the basket? The information that it's one and the same basketball traversing a single path is not given by sentience if sentience is limited to feature-placing. [According to the spatiotemporal regions view], the information that it's one and the same basketball traversing a single path has to be given non-sensorily. The subject's visual experience stops short” (Siegel, 2002, p. 137, see also Matthen, 2004, 2005, Cohen, 2004, Pylyshyn, 2007, p. x).

Third, if the spatiotemporal regions view were correct, then it would be difficult to account for perceptual justification: perception is about spatiotemporal regions, whereas beliefs that are perceptually justified are about ordinary objects. Here is Siegel again: “if audition told us that it was a place, rather than something at that place, that was cheeping, we would have all sorts of errors to correct in the move from audition to thought” (Siegel, 2002, p. 137, see also Matthen, 2012).

The first argument is about how the spatiotemporal regions view is in conflict with the way we take ourselves to be perceiving. We take ourselves to be perceiving objects, not places. But it is unclear what these considerations have to do with the debate about the nature of sensory individuals as sensory individuals are not what we take ourselves to be perceiving: taking ourselves to be perceiving a kind of entity is neither necessary nor sufficient for in fact perceiving that sensory individual. This distinction takes away the bite of first argument against the spatiotemporal regions view.

Some of the force of this way of countering the first argument carries over to the second argument as well. If the issue is that we do take ourselves to be perceiving the very same basketball when we see it on its way towards the basket, then this is, strictly speaking, irrelevant for deciding what the sensory individual is as it is a consideration about what we take ourselves to be perceiving and not about the nature of sensory individuals (as perceptual content is not directly introspectable).

But the real problem with the second argument is that the claim we are examining is that the sensory individuals of vision are spatiotemporal regions, not that they are spatial regions. The spatial region the basketball occupies changes over time. The spatial region it occupied a second ago was by my hand. The spatial region it occupies now is by the hoop. So if the claim were that the sensory individuals of perception are spatial regions, then this claim would need to be supplemented with an account of how spatial regions change. But the claim we examine is not that the sensory individuals of vision are spatial regions, but that they are spatiotemporal regions. A spatiotemporal region is by definition fixed to one time interval, but it is an open question how wide this time interval is. A spatial region can take any three-dimensional shape. Similarly, a spatiotemporal region can take any four-dimensional shape, including the shape of the exact trajectory of the basketball. In short, the spatiotemporal regions view does not exclude the possibility of the perceptual representation of movement. In fact, an important part of the visual cortex, namely, V5 (MT) is universally described as representing movement (see the end of the present subsection below for some support that these count as genuine representations), but the sensory individuals of the retinotopic V5 representations are not objects of any kind because objects don’t show up until much later in visual processing (Kolster et al., 2010; Reppas et al., 1997).

How about the third argument—the argument from perceptual justification? Here an analogy with the debate about the range of perceptually attributed properties could be helpful. No philosopher claims that the range of properties attributed perceptually is the same as the range of properties attributed (perceptually or non-perceptually). I may represent my laptop as having the property of being made in Malaysia in 2019, but this property is very unlikely to be perceptually attributed. But the justificatory transition from perception to belief implies that a new range of properties are attributed by the belief that is justified by the perceptual state that does not attribute these properties. Thus, there is a necessary mismatch between the range of properties attributed in perception and the range of properties attributed by the belief—no-one seems to worry about the possibility of perceptual justification because of this. And there is no reason to believe that things are different when we consider entities, rather than properties. If the mismatch between the perceptually attributed properties and the non-perceptually attributed properties is not worrisome for the prospects of perceptual justification, then the mismatch between the sensory individuals and the particulars our beliefs are about should not be worrisome either.

In short, the spatiotemporal region view might have been discarded prematurely. And there are also some positive considerations in its favor.

One consideration that makes spatiotemporal regions an attractive candidate for the sensory individual of vision comes from the empirical study of early vision. The first thing to note is that early vision has genuine representations, regardless of what (even remotely naturalistic) account of representation one endorses. If one takes constancies to be the mark of representations (Burge, 2010), then the primary visual cortex already represents: there are demonstrated lightness and size constancies in the primary visual cortex (MacEvoy & Paradiso, 2001; Murray et al., 2006). And if one takes the possibility of misrepresentation to be the mark of representations (Dretske, 1988), we get the same results: the visual cortices can and do misrepresent. When we look at an illusory contour, the primary visual cortex represents an edge, but the edge is not there. It misrepresents. And the same goes for the representation in V4, MT, and so on. Early cortical representations are *bona fide* representations.

Remember that sensory individuals are what properties are perceptually attributed to. Early vision attributes properties like contours, shape and color. But what does it attribute these properties to? Not to ordinary objects because ordinary object-representation is supposed to happen further down the perceptual processing.^4^ Early vision is retinotopic and features (or properties), like contours are attributed not to objects, but to spatiotemporal regions that are isomorphic to the retina. In other words, in early vision, properties like contour, shape and color properties are attributed to spatiotemporal regions (and not to ordinary objects).

As we shall see in Sect. 6, this does not mean that the ordinary object view of the sensory individual of vision in general needs to be discarded, given that vision is more than early vision. But the considerations presented in this section in defense of the spatiotemporal region view show that this view is a genuine candidate for explaining the sensory individual of picture perception.

## Spatiotemporal regions in picture perception

My claim is that when we look at pictures, we perceptually attribute properties not to particulars or object-types or appearances or to no sensory individual at all. We perceptually attribute properties to spatiotemporal regions of the pictorial space. Spatiotemporal regions are regions in time–space. They are not particulars. Hence, the argument against the claim that the sensory particulars of picture perception are particulars don’t apply here.

There are spatiotemporal regions around you, but if you look at a picture, there are also spatiotemporal regions depicted in the picture. When you look at the Mona Lisa, the spatiotemporal region depicted in that picture is disconnected from the spatiotemporal region around you in the Louvre (where this painting is located). There is no fact of the matter about the distance between you and the woman depicted in Mona Lisa, for example. When we look at this picture, we get a glimpse into a different spatiotemporal region—a spatiotemporal region that is temporally very narrow and spatially also somewhat limited (by the picture frame).

We have seen that if properties are attributed to spatiotemporal regions, this solves the Many Properties Problem and it does so for picture perception as much as it does for ordinary vision. Seeing a picture of a red square on the left and a blue triangle on the right is different from seeing a picture of a red triangle on the left and a blue square on the right.

And the spatiotemporal view can explain this difference in terms of properties attributed to the spatiotemporal region in the left side of the pictorial space and the spatiotemporal region in the right side of the pictorial space. In the first case, the properties of being red and being square-shaped are attributed to the spatiotemporal region in the left side of the pictorial space and the properties of being blue and being triangle-shaped are attributed to the spatiotemporal region in the right side of the pictorial space. And in the second case, the properties of being red and being triangle-shaped are attributed to the spatiotemporal region in the left side of the pictorial space and the properties of being blue and being square-shaped are attributed to the spatiotemporal region in the right side of the pictorial space. Clearly very different episodes of picture perception.

So when you are looking at a caricature of Mick Jagger, you perceptually attribute properties to spatiotemporal regions of the pictorial space. It is not Mick Jagger, the particular person, whom you perceptually attribute properties to. This distinction respects and even elucidates an important distinction often made in the picture perception literature. In Section I, I said that picture perception poses extra complications for pinning down the sensory individual because it is really two perceptual episodes that happen simultaneously. We see the picture surface and we also see the depicted scene. But it has long been argued that there are in fact not two but three perceptual (or quasi-perceptual) episodes that constitute picture perception (Nanay, 2018, Mion, 2018, Voltolini, 2018, see also Wiesing, 2009 on the historical antecedents of this view). We perceptually represent three things: (A) the (two-dimensional) picture surface, (B) the three-dimensional scene visually encoded by the picture surface and (C) the depicted object. Crucially, (B) and (C) are different. To return to the example of the caricature of Mick Jagger, (B) has more distorted features (thicker lips, say) than (C).

There are contentious theories about the relation between these three perceptual representations and I will not defend any specific version of these (which are often go with the label of ‘threefoldness’). My aim is to zero in on an uncontested and widely agreed on common denominator that both proponents and opponents of the threefoldness accounts (which claim, roughly, that we are simultaneously and perceptually aware of (A), (B) and (C) when looking at pictures) would agree on.^5^ So I will not make any claims about how (C), that is, the depicted object, is represented, whether it is represented perceptually, by means of mental imagery or non-perceptually.

All I need to appeal to is that the representation of (B) is different from the representation of (C). The three-dimensional scene visually encoded in the picture surface is different from the depicted scene and the representation of the former is different from the representation of the latter. In the case of many pictures, like caricatures or black and white pictures, (B) and (C) will look very different. In the Mick Jagger caricature, (B) has grossly big lips, whereas (C) does not. In a black and white photograph of an apple, (B) is some shade of grey, but (C) isn’t.

But even in high quality color photographs or photorealist paintings, (B) and (C) are very different kinds of entities. As we have seen, (C) is often (maybe even always) a particular. Mick Jagger is a particular person. The apple in the black and white photograph is a particular apple, even if it may look exactly the same as some other apple. Crucially, (B) is not a particular. (B) is the three-dimensional scene visually encoded by the picture surface. And the two-dimensional picture surface can only visually encode a spatiotemporal region and its properties. It could not visually encode a particular.

One way of seeing this is to ask about the determinacy of (B) and (C). If (C) is a particular, as in the case of photographs and portraits, its properties are superdeterminates: determinates without further determinates. The color of the apple depicted in the black and white photo is a maximally specific shade of red. But the properties of (B) are determinable. In the case of the black and white photo of the apple, (B) has some very determinable color properties, for example.

Seeing something in a picture is seeing (B). Much of the representational content of the picture is provided by (C) (which can be a particular), but what you see is (B). And much of the representational content of our perceptual state is also provided by the representation of (C), which can enrich our (in itself quite sparse) perception of (B). In the case of seeing the black and white photograph, our knowledge that apples tend to be red may literally color our experience seeing an apple in this photo (in fact, empirical findings show that this is exactly what happens, see Hansen et al., 2006).

Whether (C) is perceptually represented is very much an open question and according to most accounts of picture perception, (C) is not perceived: it is represented non-perceptually or maybe by means of mental imagery. Thus, the question about the sensory individual of picture perception is a question about the nature of (B). And, as we have seen, the most natural way of understanding (B) is that it is a spatiotemporal region.

In short, the claim that the sensory individuals of picture perception are spatiotemporal regions is very much consistent with widely shared assumptions about the kind of representational episodes that underlie picture perception. In fact, it is predicted by these minimal assumptions.

## Conclusion: a pluralistic account of sensory individuals

I argued that the sensory individuals of picture perception are spatiotemporal regions. Does it follow from this claim that we need to completely revise the debate about the sensory individuals of vision in general—and maybe also about the sensory individuals of other sense modalities? Does it mean that we can have a unified account of the sensory individuals of vision (or even of all sense modalities) as spatiotemporal regions?

I don’t think so. Perception is a complex process and at different stages of perceptual processing, different properties are attributed to different sensory individuals (maybe even using different formats). Further, perception is not all-purpose (Nanay, 2021b). We can use our perceptual abilities in different ways and for different purposes and these may vary not only in what kind of properties we attribute perceptually, but also in what these properties are attributed to.

The conclusion then is a pluralistic one: we need to talk about the diversity of sensory individuals (this would be a much broader diversity than the one considered in Matthen, 2010 for the auditory sense modality). Some sensory individuals are ordinary objects. Some are spatiotemporal regions. And some are sounds or odors. Crucially, the best candidate for the sensory individuals of picture perception are spatiotemporal regions.

## Footnotes

1 I will use the example of the depicted apple in this subsection, which is an ordinary object, but note that the same argument would apply if I talked about events, not objects (as both objects and events are supposed to be particulars, which is really what the arguments below turn on).

2 Clark’s concept of ‘phenomenal individual’ may be narrower than the concept of sensory individual (as the former, but not the latter seems to imply that the perceptual representation in question is conscious. See Clark (2004) for discussion.

3 I am bracketing supersubstantivalism here, but even some versions of supersubstantivalism would be consistent with the claim that spatiotemporal regions are not particulars (see Lehmkuhl, 2018).

4 Even fans of ‘object files’ (I’m not one of them) would admit this as object files are supposed to show up only in mid-level vision.

5 The distinction between (B) and (C) (or something very much like it) is widespread in the literature, even among those who explicitly argue against threefoldness accounts. See, for example, Rob Hopkins’s distinction between’seeing-in content’ and ‘pictorial content’ (Hopkins, 1998, p. 128) or Catharine Abell’s distinction between internal and external objects (Abel,l 2010, esp. pp. 83ff, see also Abell, 2009).
